# VCAM1 Promotes Tumor Cell Invasion and Metastasis by Inducing EMT and Transendothelial Migration in Colorectal Cancer

**DOI:** 10.3389/fonc.2020.01066

**Published:** 2020-07-23

**Authors:** Dan Zhang, Jiaxin Bi, Qiaoyan Liang, Shuyang Wang, Lingjie Zhang, Fangyi Han, Shengnan Li, Bowen Qiu, Xingdi Fan, Wei Chen, Hongli Jiao, Yaping Ye, Yanqing Ding

**Affiliations:** ^1^Department of Pathology, School of Basic Medical Sciences and Nanfang Hospital, Southern Medical University, Guangzhou, China; ^2^Guangdong Provincial Key Laboratory of Molecular Tumor Pathology, Guangzhou, China

**Keywords:** VCAM1, EMT, TEM, metastasis, colorectal cancer

## Abstract

Vascular cell adhesion molecular 1 (VCAM1), an important member of the immunoglobulin superfamily, is related to the development of malignant tumors, such as breast cancer, melanoma, and renal clear cell carcinoma. However, the molecular role and mechanism of VCAM1 in the regulation of the progression of colorectal cancer (CRC) has rarely been studied. The results of IHC and RT-PCR analyses proved that VCAM1 was upregulated in human CRC tissues compared with matched adjacent normal intestinal epithelial tissues. Moreover, analysis of data from the TCGA and Gene Expression Omnibus (GEO) databases revealed that a higher level of VCAM1 was strongly correlated with poor differentiation, metastasis, and short survival in CRC patients. Furthermore, VCAM1 significantly influenced the invasion and metastasis of CRC cells *in vitro* and *in vivo* and activated the EMT program, by which cancer cells adhere to the endothelium and cross the vessel wall by forming pseudopodia and invadopodia. The current findings demonstrate that VCAM1 promotes tumor progression in CRC.

## Introduction

Colorectal cancer (CRC) is the third most common malignant cancer worldwide (1–3). Surgery, chemotherapy, and radiotherapy remain fairly effective for treating various kinds of non-metastatic tumors, but these routine therapeutic methods fail to restrict the development of metastasis once it occurs. Thus, colorectal cancer metastasis remains a critical factor in cancer-related death (3, 4). Colorectal carcinogenesis is a process with multiple steps that are associated with complex genetic and epigenetic alterations (3). It is necessary to identify efficient molecular markers and therapeutic targets.

Vascular cell adhesion molecular 1 (VCAM1) protein, a member of the immunoglobulin superfamily containing seven extracellular Ig domains, is generally detected in endothelial cells and activated by inflammatory factors (5); VCAM1 is involved in the occurrence and development of inflammatory disease (6–8), especially in the process of trans-endothelial migration. It is now well-established from a variety of studies that VCAM1 is closely related to the development of malignant tumors, such as breast cancer (9), ovarian cancer (10), and clear cell renal carcinoma (11). Furthermore, researchers discovered that tumor cells' overexpression of VCAM1 accomplished lung or bone metastasis by recruiting monocytes or macrophages and forming a complex that facilitates circulating tumor cell evasion from immune system attack and trans-endothelial migration (12). However, far too little attention has been paid to the clinicopathological expression pattern and functional role of VCAM1 in the progression of CRC.

Cancer metastasis is the product of a multistep, continuous cell-biological response process known as the invasion-metastasis cascade, which depends on epithelial-mesenchymal transformation (EMT), in which tumor cells lose their polarity and cell adhesion and become mesenchymal cells with strong migration and invasion abilities (13). EMT induces the adhesion of tumor cells to endothelial cells and transendothelial migration (TEM), in which cell adhesion molecules (CAMs) play an important role (14). In colorectal cancer, the downregulation of some CAMs (such as cadherins, catenins, integrins, and selectins) promotes the progression of TEM to metastasis via enhancing tumor cell adhesion to the surrounding interstitium (15). As an important member of CAMs, however, the role of VCAM-1 in the development of colorectal cancer is still unclear.

This study aimed to investigate the expression pattern and functional role of VCAM1 in the tumorigenesis and metastasis of CRC, and to explore the molecular mechanism by which VCAM1 regulates invasion and metastasis. The findings of this research provide insights for new potential therapeutic strategies and targets for patients with CRC.

## Materials and Methods

### Bioinformatics Analysis

GSE17536, GSE17538 datasets were downloaded from the GEO database and were used to evaluate the effect of VCAM1 on overall patient survival. GSE41258 and TCGA database analysis were performed to discover the enrichment of signaling pathways for VCAM1. GEPIA was used to analyze the correlation between VCAM1 and key genes of the EMT program.

### Clinical Tissue Specimens

CRC tissues and matched adjacent normal tissues (n = 123) were collected by surgical resection from patients with primary colorectal adenocarcinoma at Southern Hospital of Southern Medical University (Guangzhou, China), and none of them received radiotherapy or chemotherapy before surgical removal. Fifteen samples were frozen in liquid nitrogen for qPCR.

### RNA Isolation, Reverse Transcription (RT), and Real-Time Quantitative PCR

Total RNA from all cell lines and tissues were isolated using the Trizol reagent (Invitrogen, CA, USA) according to the manufacturer's instruction. RT was carried out with the SuperScript First-Strand Synthesis System for RT-PCR (Invitrogen, CA, USA) according to the protocol. Primers were designed using Primer 5.0 software. The primers used for the PCR were as follows: VCAM1: 5′-GTCTCCAATCTGAGCAGCAA-3′ (forward) and 5′-TGGGAAAAACAGAAAAGAGGTG (reverse); GAPDH: 5′-ACAGTCAGCCGCATCTTCTT-3 (forward) and 5′-GGATGCCACAGGACTCCAT-3′ (reverse). Real-time quantitative PCR was performed using the Applied Biosystems 7500 Sequence Detection system, using iQ™ SYBR Green Supermix (Bio-Rad Laboratories, Hercules, CA, USA) containing 5 ng cDNA and 10 pm of each primer. The cycling conditions were: one cycle at 94°C for 5 min; 40 cycles of 95°C for 30 s, 56°C for 30 s. Melting curve analysis was carried out for each PCR reaction to confirm the specificity of amplification. Real-time quantitative PCR for target genes was performed as previously described (16). The data were normalized to the geometric mean of the housekeeping gene GAPDH and calculated as 2^−ΔΔ*CT*^ method.

### Western Blotting

Briefly, equal amounts of protein were separated by electrophoresis on a 10% sodium dodecyl sulfate polyacrylamide gel and electrotransferred from the gel to a nitrocellulose membrane. After blocking with 5% BSA solution in Tris-buffered saline with Tween (TBS-T) for 1 h, the membrane was incubated with primary antibody against rabbit antibody Anti-VCAM1(Abcam, Cambridge, MA, USA), anti-E-cad, anti-N-cad, anti-ZEB, anti-ZO1, anti-MMP9, anti-Snai1, anti-Vimentin, anti-Rac1, anti-Cortactin, anti-LIMK, anti-Cofilin, anti-CDC42, and anti- PAK (Cell Signaling Technology, Danvers, MA, USA) overnight at 4°C. A mouse anti-α-Tubulin monoclonal antibody (Sigma, Saint Louis, MO, USA) was used as a loading control. After washing with TBS-T, the membrane was incubated with a secondary antibody against rabbit immunoglobulin G or mouse immunoglobulin G (Ray Antibody Biotech, BeiJing, China); then, it was examined with the enhanced chemiluminescence detection system (Yeasen, ShangHai, China) according to the manufacturer's protocol.

### Tumor-Endothelial Cell Adhesion Assay

HUVECs (the American Type Culture Collection, Manassas, VA, USA) were labeled with green fluorescent protein GFP. HUVECs transiently transfected with pLenti-EF1α-GFP-Flag-puro plasmid (Vigenebio, Shandong, JN, China) using the Lipofectamine® 2000 (Invitrogen, Carlsbad, CA, USA) were seeded in 96-well plates, then allowed to grow to confluence and stimulated with human TNF-α (10 ng/ml) (Cell Signaling Technology, Danvers, MA, USA) for 12 h. Tumor cells labeled with red fluorescent protein mCherry (Vigenebio, Shandong, JN, China) were added to the endothelial cell monolayer for 30 min. Later, the 96-well plate was washed with PBS three times to remove non-adherent cells. The average number of adherent cells was calculated by using a fluorescence microscope (LSM 880 with Airyscan). Each condition had three replicate wells.

### Cancer Cell Transendothelial Migration Assay

Approximately 1 × 10^5^ HUVECs were seeded in the upper chamber of a Transwell insert and allowed to grow to confluence. Then, the monolayer of cells was treated with human TNF-α (10 ng/ml) for 12 h. The medium was removed, and tumor cells labeled with red fluorescence mCherry were added on top of the HUVEC monolayer. Medium (1640) with 20% FBS (Gibco, Grand Island, NY, USA) was used as a chemoattractant. Cells were allowed to migrate for 48–72 h at 37°C in 5% CO_2_. The number of cells that migrated to the basolateral side of the Transwell membrane was calculated by using a fluorescence microscope.

### Scanning Electron Microscopy to Observe the Formation of Pseudopodia

Cover slips were put into the 24-well plate placed on the ice and then 200 μl of Matrigel (BD biosciences, New York, USA) per well was quickly added. After solidification at 37°C in humidified air with 5% CO_2_ for 30 min, 2 × 10^5^ cells with VCAM1 overexpression or knockdown were added. The cells were washed with PBS three times after being cultured for 36 h. Cells were fixed with 2.5% glutaraldehyde for 2–4 h, then washed twice with PBS. Cells were taken to a scanning electron microscopy (Hitachi, S-3000N) specialist to carry out the rest of the procedure. Finally, we obtained images of the cells with different magnifications.

### Mouse Experiments

Four- to six-week-old Balb/C athymic nude mice that were raised under SPF conditions were obtained from the Animal Center of Southern Medical University, Guangzhou, China. All mouse experiments were carried out in accordance with Committee for the Care and Use of Animals and proceeded on the basis of institutional guidelines. RKO cells expressed with stable VCAM1-overexpressing or scramble control and HCT116 cells expressed with stable VCAM1 knockdown cells or scramble control shRNA (5 × 10^6^, *n* > 3 for each group) were injected into spleen capsules and tail veins of nude mice separately to build the liver and lung metastasis model. Mice were euthanized on the 20th day. The number of metastases in the lung and liver was observed by the naked eye and recorded. Tumors were fixed, and 4 μm sections were cut. Hematoxylin and eosin (HE) staining was performed according to standard protocols.

### Statistical Analysis

All statistical data were performed with SPSS version 20.0. Quantitative data are presented as the mean ± SEM. Statistical analyses included Student's *t*-test, Wilcoxon-Mann-Whitney test, chi-square test for contingency tables, or one-way ANOVA. All experiments were performed three times, and the data from a single representative experiment are presented. Survival curves were plotted by the Kaplan-Meier method and compared using the log-rank test. *P* < 0.05 was considered significant.

For other methods, please see the Supplementary Data.

## Results

### Upregulation of VCAM1 Is Associated With Aggressive Characteristics of CRC

**Figure 1:**
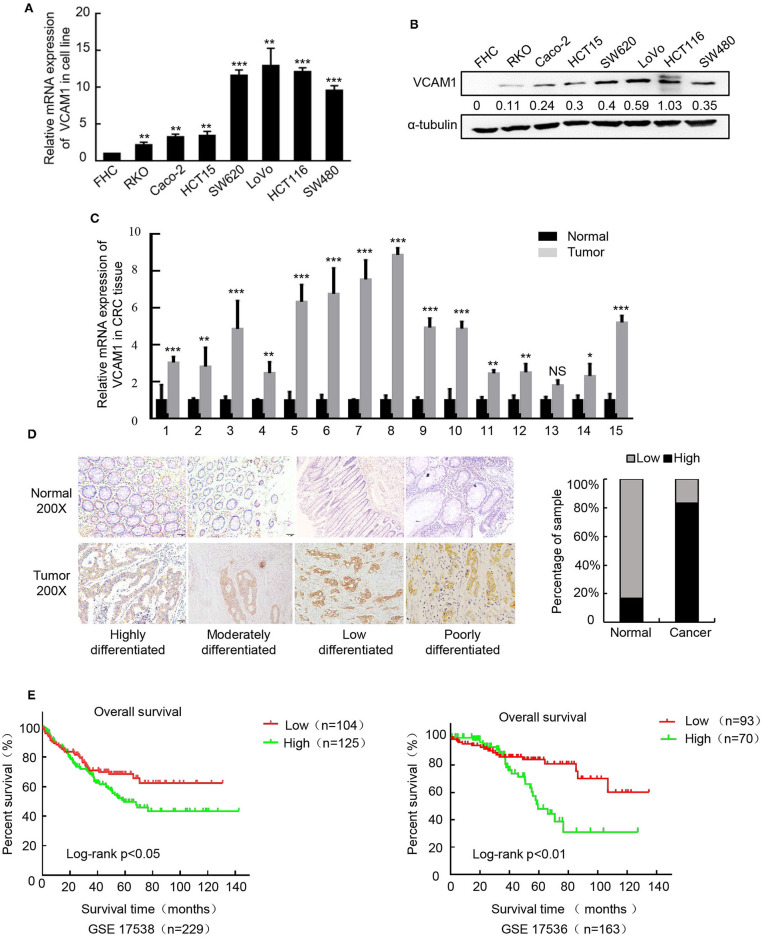
Upregulation of VCAM1 is associated with aggressive characteristics of CRC. **(A,B)** The protein and mRNA expression of VCAM1 by western blotting and Real-time PCR in CRC cell lines. α-tubulin or GAPDH were used as internal reference. **(C)** Expression of VCAM1 mRNA by real-time PCR in 15 CRC and matched specimens. **(D)** Representative images of VCAM1 staining in CRC tissues and adjacent normal tissues (*n* = 123) evaluated by IHC. Scale bars: 50 μm. Histograms indicate the statistics of VCAM1 expression. **(E)** Correlation of VCAM1 expression and overall survival by Kaplan-Meier analysis in CRC patients from a public clinical microarray database, GSE17538 (left) and GSE17536 (right). Error bars represent the mean ± *SD* calculated from three parallel experiments. *p < 0.05, **p < 0.01, ***p < 0.001.

To confirm the expression pattern of VCAM1 involved in the progression of CRC, real-time PCR and Western blotting were performed in 8 cell lines, including FHC (normal colon mucosa), RKO, Caco-2, HCT15, SW620, LoVo, HCT116, and SW480, and the results showed obviously higher levels of VCAM1 mRNA and protein in human colorectal cancer cells than in normal colon mucosa cells (Figures 1A,B). In addition, VCAM1 mRNA levels were also higher in 15 fresh CRC tissues compared with paired adjacent normal intestine tissues (Figure 1C). On the other hand, we analyzed the expression of VCAM1 in 123 paraffin-embedded CRC tissues by IHC. A total of 86 CRC tissues (70%) exhibited high expression of VCAM1 compared to their matched adjacent normal tissues (Figure 1D). VCAM1 was mainly observed in the cytoplasm and cell membrane of epithelial cells (Figure 1D). In addition, the expression levels of VCAM1 are positively correlated with variously differentiated colorectal cancer (Figure 1D). Concerning the correlation between VCAM1 expression level and clinicopathological parameters, we analyzed the detailed data. The chi-square test analysis demonstrated that up-regulation of VCAM1 expression was significantly associated with more aggressive tumor phenotypes, such as poor differentiation (*P* < 0.0001) and more distant metastases (*P* = 0.0271) (Table 1). In addition, Univariate and Multivariate analyses of various prognosis parameters in 123 CRC patients were used; the results showed that VCAM1 expression was a significant prognostic factor (Supplementary Table S1). Survival data from the public database were analyzed by the Kaplan-Meier method, and the curve showed that the survival time of patients with high VCAM1 expression was shorter than that of those with low VCAM1 expression (Figure 1E). From the above findings, this investigation showed that increased VCAM1 expression was significantly associated with a more invasive tumor phenotype and a poorer prognosis in CRC patients.

**Table 1:** Relationship between VCAM1 expression and CRC clinicopathological parameters.

**Clinicopathological features**	***n***	**VCAM1 expression**		***χ^2^* value**	***P-*value**
		**Low**	**High**		
**Age**					
<60	63	21	42	0.524	0.4689
≥60	60	20	40		
**Gender**					
Male	69	26	43	1.143	0.2851
Female	54	15	39		
**Differentiation**					
Well	83	32	51	17.98	<0.0001
Moderate/poor	40	9	31		
**TNM stage**					
I/II/III	102	35	67	6.838	0.0089
IV	21	6	15		
**T classification**					
Tis	4	4	0		
T1/T2	28	9	19	1.146	0.284
T3/T4	91	28	63		
**N classification**					
N0	83	30	53	1.754	0.1854
N1/N2	40	11	29		
**M classification**					
M0	102	35	67	4.886	0.0271
M1	21	6	15		

### VCAM1 Positively Regulates the Invasion and Metastasis of CRC Cells *in vitro* and *in vivo*

To explore the potential role of VCAM1 in the progression of CRC, we chose to use RKO and Caco-2 to generate stable VCAM1-overexpressing cell lines, while LoVo and HCT116 were used to generate VCAM1 knockdown cell lines. Real-time PCR and Western blotting confirmed the high transfection efficiency (Supplementary Figures 1A–D). First, we observed the growth and proliferation of the four CRC cell lines. MTT assays, plate colony formation assays, and subcutaneous xenotransplanted tumor models were used, and no significant differences were found between cells with altered VCAM1 expression and control cells *in vivo* and *in vitro* (Supplementary Figures 2A–F). These data suggested that VCAM1 does not regulate proliferation in CRC cells.

**Figure 2:**
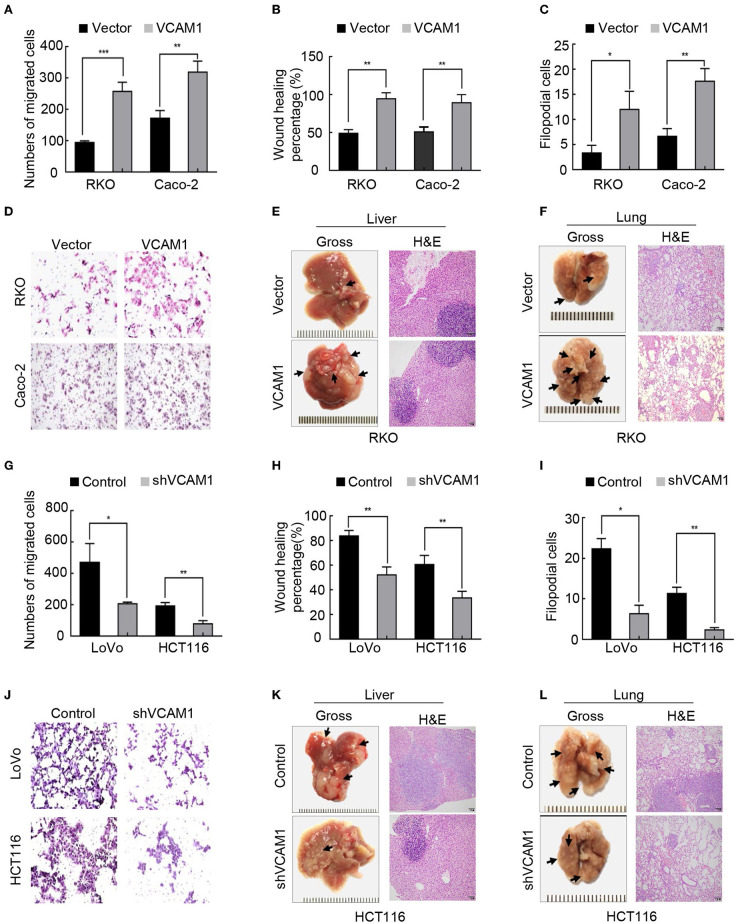
VCAM1 positively regulates the invasion and metastasis of CRC cells *in vitro* and *in vivo*. **(A–C)** Histograms showing cell migration by Transwell assays **(A)** and wound healing assays **(B)** and cell invasion by 3D cell culture assays **(C)** in RKO and Caco-2 cells overexpressing VCAM1. **(D)** Detection of cell invasion using Matrigel-coated Transwell assays in the indicated CRC cells. **(E,F)** Images of metastasis in the lung and liver after injecting RKO cells with high levels of VCAM1 into the tail vein and spleen capsule of nude mice (*n* > 3). Gross observation (left) and metastatic foci by HE staining (right). **(G–I)** Quantification of cell migration via Transwell assays **(G)** and wound healing assays **(H)** and cell invasion by 3D cell culture assays **(I)** in LoVo and HCT116 cells with depletion of VCAM1. **(J)** Detection of cell invasion via Matrigel-coated Transwell assays in the indicated CRC cells. **(K,L)** Establishment of lung and liver metastatic carcinoma model in which nude mice (*n* = 5) were injected with HCT116-vector and HCT116-shVCAM1 cells through the tail vein and spleen capsule. Quantification of the number of metastatic foci by gross observation (left) and HE-stained images (right). Scale bars: 50 μm. Error bars represent the mean ± *SD* from three independent experiments. *p < 0.05, **p < 0.01, ***p < 0.001.

Then, we examined migration and invasion in the four CRC cell lines. The results of the Transwell assay and wound healing assay showed that overexpression of VCAM1 in RKO and Caco-2 cells enhanced cell migration ability (Figures 2A,B, Supplementary Figures 3A,B). Furthermore, the results of 3D cell culture and Matrigel-coated Transwell assays showed that overexpressing VCAM1 in RKO and Caco-2 cells effectively increased the invasive abilities (Figures 2C,D, Supplementary Figures 3C,D). To estimate the influence of VCAM1 on CRC metastasis *in vivo*, lung and liver metastasis models were established. The number of metastatic nodules in the livers and lungs of nude mice injected with VCAM1-overexpressing cells was notably increased compared with that in those injected with control cells (Figures 2E,F, Supplementary Figures 3E,F). In contrast, suppression of VCAM1 in LoVo and HCT116 cells showed the opposite effect: VCAM1 depletion lowered the invasion and metastasis ability of specific CRC cell lines *in vivo* and *in vitro* (Figures 2G–L, Supplementary Figures 3G–L).

### VCAM1 Promotes Invasion and Metastasis via Activating EMT in CRC Cells

**Figure 3:**
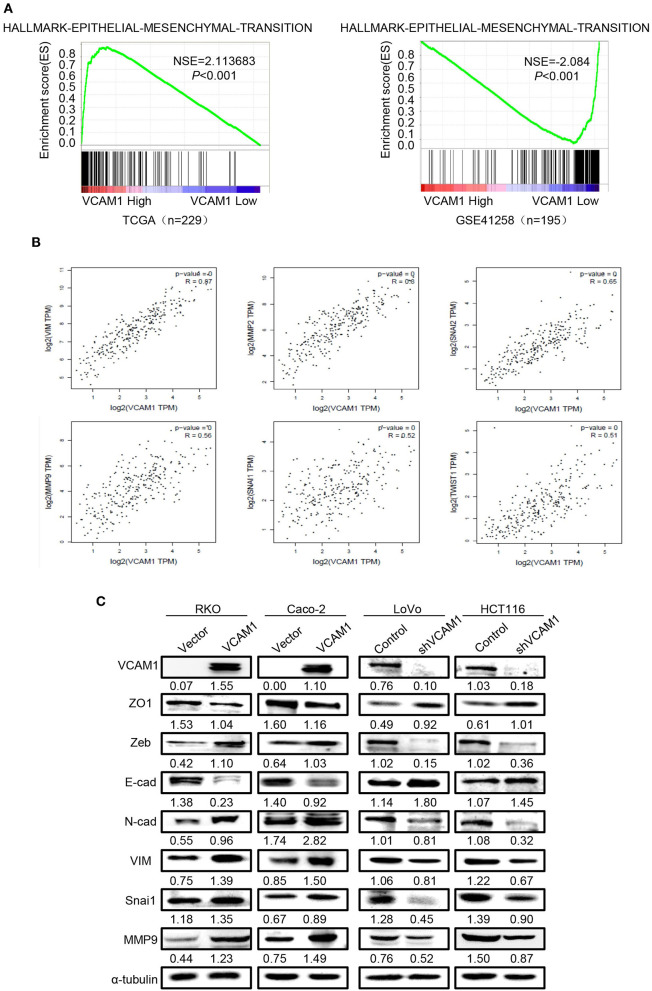
VCAM1 promotes invasion and metastasis via activating EMT in CRC cells. **(A)** TCGA (*n* = 229) and GSEA (GSE41258, *n* = 195) analyses of the published CRC gene expression database were used to analyze the VCAM1-regulated gene signatures. **(B)** GEPIA was used to analyze the interaction between VCAM1 and key genes of the EMT program. **(C)** The expression of VCAM1, ZO1, Zeb, E-cad, N-cad, VIM, Snai1, and MMP9 in the indicated cells was detected by Western blotting.

GSEA and TCGA were used to analyze the VCAM1-regulated gene signatures. The results of TCGA and GSEA(GSE41258) analysis revealed that high expression of VCAM1 was positively correlated with the enrichment of the EMT program (Figure 3A). Meanwhile, GEPIA (Gene Expression Profiling Interactive Analysis) (17) was used to analyze the interaction between VCAM1 and key genes of the EMT program, which suggested VCAM1 was highly correlated with key genes of the EMT pathway (Vimentin, MMP2, Snai2, MMP9, Snai1, Twist1) (Figure 3B). These results indicate the involvement of VCAM1 in the regulation of EMT.

There is no denying that the activation of EMT is a key process by which cancer cells leave the primary tumor site and fulfill the process of migration and invasion (18, 19). As shown in Figure 3C, the expression of mesenchymal markers, such as N-cad, Vimentin, Snai1, Zeb, and MMP9, was upregulated, while epithelial markers, such as E-cad and ZO1, were reduced in CRC cells overexpressing VCAM1. Furthermore, we verified the result in the opposite way in CRC cells with VCAM1 knockdown (Figure 3C). The analysis above means that VCAM1 participates in regulating the EMT process of CRC cell lines. In conclusion, these results imply that VCAM1 is involved in the induction of EMT in CRC.

### VCAM1 Plays a Positive Role in the Progression of TEM in CRC Cells

**Figure 4:**
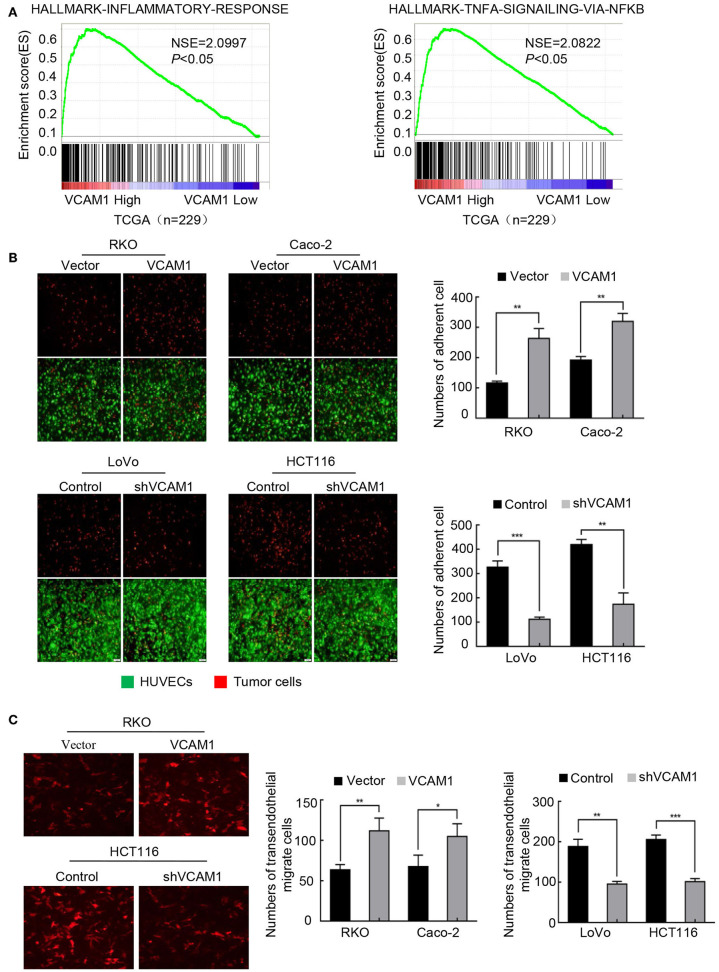
VCAM1 plays a positive role in the progression of TEM in CRC cells. **(A)** TCGA (*n* = 229) enrichment analysis was adopted to analyze the VCAM1-related signatures. **(B)** Evaluation of CRC cell adhesive activity by tumor-endothelial cell adhesion assay in cells with VCAM1 upregulation (above) and downregulation (below). Red: tumor cells, green: HUVECs. Histogram analysis of the numbers of adherent cells (right). Scale bars: 50 μm. **(C)** Detection of TEM activity by transendothelial migration assay in the indicated cells (red). Scale bars: 50 μm. Histogram analysis of the numbers of transendothelial migrate cells (right). Error bars represent the mean ± *SD* from three independent experiments. *p < 0.05, **p < 0.01, ***p < 0.001.

Tumor cells adhere to the endothelium, which is a necessary step for migration into blood vessels. Recent evidence suggests that VCAM1 interacts with VLA4 on endothelial cells, which mediates tumor cell adhesion, vascular extravasation, and brain metastases formation in breast cancer (20) and induces the formation of an inflammatory environment (10, 21). TCGA enrichment analysis revealed that the inflammatory signature and TNF-α signaling were enriched in the high VCAM1 expression group (Figure 4A). We considered the possibility that VCAM1 could affect the adhesion ability of CRC cells. As expected, a tumor-endothelial cell adhesion assay identified that forced expression of VCAM1 in RKO and Caco-2 cells caused a significant increase in the ability of these cells to adhere to HUVECs (Figure 4B), while suppression of VCAM1 impaired the adhesion of LoVo and HCT116 cells (Figure 4B).

TEM is a dynamic activity that involves the secretion of various kinds of cytokines by tumor cells, destroying cadherins among endothelial cells (22). Thus, we examined whether VCAM1 could regulate TEM in CRC cells. We established a Matrigel-coated Transwell assay on which HUVECs were cultured to evaluate the TEM capability of CRC cells by calculating the number of cells on the basolateral side of the Transwell membrane. As we expected, the results showed that, compared with the control cells, more RKO and Caco-2 cells with upregulation of VCAM1 were found on the subsurface of the Transwell membrane under a fluorescence microscope (Figure 4C). In contrast, depletion of VCAM1 in HCT116 and LoVo cells decreased the TEM ability (Figure 4C).

### VCAM1 Promotes the Formation of Pseudopodia in CRC Cells

**Figure 5:**
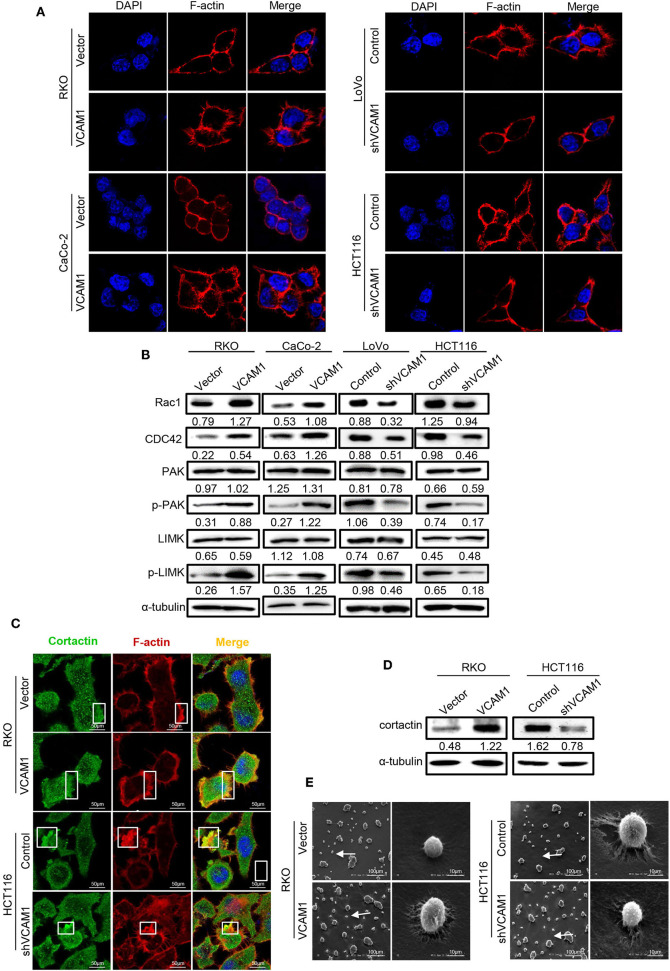
VCAM1 promotes the formation of pseudopodia in CRC cells. **(A)** F-actin was stained with rhodamine phalloidin (red), and nuclei were stained with DAPI (blue) to evaluate the formation of pseudopodia in the indicated cells. Scale bars: 10 μm. **(B)** The expression of Rac1, CDC42, PAK, phosphorylated PAK, LIMK, and phosphorylated LIMK in the indicated cells was detected by Western blotting. **(C)** Detection of the formed invadopodia in the indicated cells. Cortactin protein was stained green, F-actin was stained for rhodamine phalloidin (red), and nuclei were stained with DAPI (blue). Scale bars: 10 μm. **(D)** Western blotting analysis of cortactin in the indicated cells. **(E)** Scanning electron microscope images of pseudopodia of cells with different VCAM1 expression. Scale bars represent 100 μm or 10 μm.

In the process of migration, tumor cells could project pseudopodia by changing the cytoskeleton to stabilize their adhesion to the endothelium and resist mechanical damage caused by the sheer force of blood flow (23). To test whether endogenous expression of VCAM1 is associated with pseudopodia formation, F-actin was labeled with phalloidin (red) in CRC cells. The images revealed that RKO and Caco-2 cells with high levels of VCAM1 exhibited more membrane protrusions than control cells. Correspondingly, we obtained the opposite results in LoVo and HCT116 CRC cells, in which the suppression of VCAM1 reduced the occurrence of pseudopodia (Figure 5A, Supplementary Figure 4A). Furthermore, we detected the expression of proteins associated with pseudopodia formation, and the results showed that ectopic VCAM1 expression upregulated the levels of Rac1, CDC42, p-LIMK, and p-PAK, while depletion of VCAM1 showed the opposite effect (Figure 5B).

Invadopodia, a type of pseudopodia, are composed of actin-rich membrane protrusions created by invasive cancer cells and contribute to the delivery and aggregation of active MMPs at sites of matrix degradation (24, 25). To assess the role of VCAM1 in invadopodia formation in CRC cells, we investigated the colocalization of F-actin with actin-bundling protein cortactin (26). In RKO cells with high levels of VCAM1, more invadopodia (~25%), existing as rosettes, were formed in comparison to control cells (~10%), while HCT116 cells with low levels of VCAM1 formed fewer invadopodia (~22%) than control cells (~40%) (Figure 5C, Supplementary Figure 4B). In addition, we investigated the expression of cortactin, which is the key protein of invadopodia formation (16), using Western blotting and found that VCAM1 positively regulated the levels of cortactin (Figure 5D). In addition, we performed scanning electron microscopy to observe the invadopodia that contributed to the degradation of the extracellular matrix (ECM), and the results showed that VCAM1 expression led to larger areas of enzymatic degradation of the focal Matrigel on the ventral side of RKO cells, while depletion of VCAM1 in HCT116 cells reduced matrix degradation (Figure 5E). These results further support the idea that VCAM1 is a facilitative factor for the formation of invadopodia in CRC cells.

## Discussion

VCAM1 is a 90-kDa glycoprotein belonging to the immunoglobin superfamily and was first identified as an endothelial cell surface glycoprotein (27, 28). Prior studies that have noted the importance of VCAM1 mainly focused on vascular inflammatory diseases and immune diseases (7), such as atherosclerosis, myocardial infarction (7), idiopathic pulmonary fibrosis (6), and rheumatoid arthritis (8). Recently, an increasing number of clinical data proved that VCAM1 is abnormally expressed in gastric cancer, renal clear cell carcinoma, melanoma, breast cancer, glioma, and other malignant tumors, and is negatively correlated with the prognosis of patients (29). However, little was found regarding the expression pattern of VCAM1 during the progression of CRC. In this study, we provided the first evidence of VCAM1 upregulation in CRC tissues compared with adjacent normal tissues. Overexpression of VCAM1 was closely associated with the invasive and aggressive clinical characteristics and poor prognosis of CRC patients.

We affirmed that there was a significant positive correlation between VCAM1 and CRC progression. A great amount of evidence has shown that VCAM1 is significantly implicated in the metastasis of cancer cells. VCAM1 was overexpressed in lung metastasis compared to primary breast cancer and mediated bone metastasis in mouse and human breast cancer cell lines (30, 31). This study confirmed that the enforced expression of VCAM1 increased the migration and invasion ability of cancer cells *in vitro* and obviously generated more and larger metastatic nodules in the lung and liver than did control cells. Interestingly, abnormal expression of VCAM1 had no effect on CRC cell proliferation.

Aggressive tumors are generally associated with activation of the EMT program, a fundamental process in cancer invasion and metastasis (32). Interestingly, public databases from the GEO and TCGA certified that expression of VCAM1 was associated with the induction of EMT, which was further validated via GEPIA analysis. In addition, this study revealed that VCAM1 promoted the development of EMT by upregulating the expression of epithelial markers and downregulating the levels of mesenchymal markers. The results suggest that the increased expression of VCAM1 conferred a higher invasive potential in CRC cells by inducing EMT.

In addition, we showed that VCAM1 regulates the process of TEM in CRC cells. In the progression of invasion and metastasis, it is necessary for tumor cells to cross the endothelial barrier and perform transendothelial migration (33, 34). Structurally, VCAM1 contains Ig-like domains 1 and 4, which are a major functional area where integrins, including α4β1 and α4β7 (27, 35), mediate leukocyte rolling and attach to the endothelium, and transmigrate through blood vessels (36, 37). Functionally, recent evidence has demonstrated the importance of VCAM1-integrin interactions in tumor metastasis in an inflammatory environment (9, 38). Surprisingly, the results of the TCGA data analysis showed that high levels of VCAM1 had a strong relationship with the inflammatory response. Subsequently, the results of the tumor-endothelial cell adhesion assay supported that CRC cells upregulating VCAM1 exhibited stronger adhesion to HUVECs. Our further study proved that VCAM1 could stimulate the TEM of colorectal cancer cells, as detected by a Matrigel-coated Transwell assay.

When tumor cells adhered to endothelial cells, the morphology of the cell membrane changed, and then pseudopodia were formed, which can help tumor cells change morphology and pass through the expanding endothelial space in the course of TEM (39, 40). The immunofluorescence results showed that overexpression of VCAM1 increased the occurrence of F-actin-rich membranous protuberance. Moreover, we found that pseudopodia formation-related proteins can also be upregulated by VCAM1 in CRC cells. As a type of pseudopodia, invadopodia are a critical construction in the process of TEM. When silencing cortactin and Tks5, the markers of invadopodia formation and functional maturation, the extravasation rate of tumor cells were decreased significantly (39). Invadopodia were also observed to be the central procedure of intravasation and extravasation (41). Our work clearly proposes that VCAM1 induced an obvious increase in the colocalization of F-actin and cortactin and matrix degradation in CRC cells.

**Figure 6:**
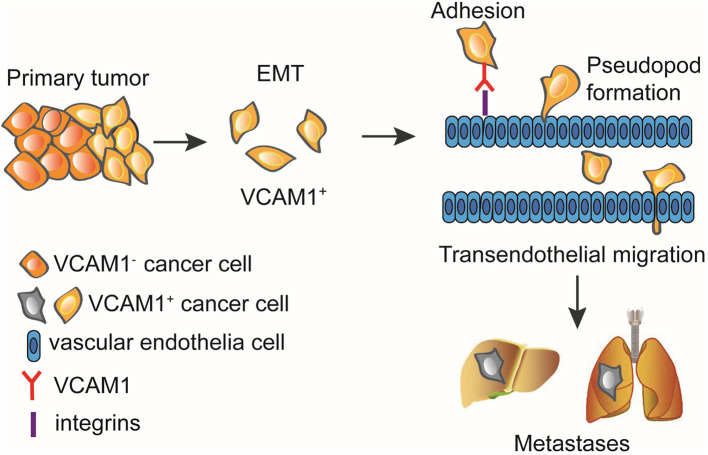
VCAM1 promotes tumor cell invasion and metastasis by inducing EMT and transendothelial migration in colorectal cancer. VCAM1 significantly influenced the invasion and metastasis of CRC cells and activated the EMT program by which cancer cells adhere to the endothelium and cross the vessel wall with the formation of pseudopodia and invadopodia.

In summary, our study demonstrates that VCAM1 improves tumor progression in CRC. Furthermore, we emphasize the regulatory mechanisms by which VCAM1 promotes invasion and metastasis of CRC via activating the EMT program, then adhering to the endothelium, and finally facilitating transendothelial migration, which is accompanied by the formation of pseudopodia (Figure 6).

## Data Availability Statement

The datasets generated for this study are available on request to the corresponding author.

## Ethics Statement

This study was carried out in accordance with the principles of the Basel Declaration and recommendations of Chinese Medical Ethics, Medical Ethics Committee of NanFang Hospital of Southern Medical University. The protocol was approved by the Medical Ethics Committee of NanFang Hospital of Southern Medical University.

## Author Contributions

DZ, JB, and QL carried out experiments. SW and HJ took on the statistical analysis. LZ, WC, and XF were mainly responsible for collecting tissue samples. FH, SL, and BQ participated in part of the animal experiments. YD and YY conceived the experiments and analyzed data. All authors were involved in writing the paper and had final approval of the submitted and published versions.

## Conflict of Interest

The authors declare that the research was conducted in the absence of any commercial or financial relationships that could be construed as a potential conflict of interest.
