# Supplementary material for: VCAM1 Promotes Tumor Cell Invasion and Metastasis by Inducing EMT and Transendothelial Migration in Colorectal Cancer
Source: Front Oncol. 2020 Jul 23;10:1066. doi: 10.3389/fonc.2020.01066 (PMC7390920; doi:10.3389/fonc.2020.01066)
Supplement: Supplementary file 1 [file Data_Sheet_1.docx]

Supplementary data

# Supplementary Materials and Methods

**Cell Culture**

Cell lines FHC, RKO, Caco-2, HCT15, SW620, LoVo, HCT116, SW480 and human umbilical vein endothelial cell (HUVEC) were purchased from the American Type Culture Collection (Manassas, VA, USA). FHC and HUVEC were cultured in DMEM (C11995500BT, Gibco, Grand Island, NY, USA) with 10% fetal bovine (10099-141, Gibco, Grand Island, NY, USA), the rest of cell lines were cultured in 1640 (C11875500BT, Gibco, Grand Island, NY, USA)) with 10% fetal bovine. All of them were cultured at 37℃ in humidified air with 5% CO_2_.

**Establishment of stable cell lines**

VCAM1 lentivirus was purchased from Obio Technology (Shanghai, China), the plasmid is plenti-CMV-Mcherry-3Flag-PGK-puro. shRNA lentivirus was purchased from Vigenebio (Shandong, JN, China), the plasmid is plenti-U6-RFP-puro. Luciferin lentivirus was purchased from Vigenebio (Shandong, JN, China). The experiment was performed according to lentivirus manual and verified by real-time qPCR and western blot.

**Immunohistochemistry**

Paraffin-embedded CRC tissues were cut into 4-µm sections and baked at 65°C for 1 hour. The sections were deparaffinized with xylenes (Guangzhou Chemical Reagent Factory, Guangzhou, China) and rehydrated. After treatment with 3% hydrogen peroxide (ZSGB, BeiJing, China) to quench the endogenous peroxidase activity, the sections were submerged into citrate buffer and high-pressure boiled for antigenic retrieval, followed by incubation with goat serum (ZSGB, BeiJing, China) to block the nonspecific binding for 30 min. Rabbit anti-VCAM1 (1:100; Abcam, Cambridge, MA, USA) was incubated with the sections overnight at 4°C. For negative controls, the rabbit anti-VCAM1 antibody was replaced with normal goat serum. After washing, the tissue sections were treated with biotinylated anti-rabbit secondary antibody (ZSGB, BeiJing, China), followed by further incubation with streptavidin-horseradish peroxidase complex (ZSGB, BeiJing, China). The tissue sections were incubated with 3,3-diaminobenzidin (ZSGB, BeiJing, China) and counterstained with hematoxylin (ZSGB, BeiJing, China), dehydrated, and mounted. The sections were reviewed and independently scored by two observers that scored the proportion of positively stained tumor cells and the intensity of staining. The proportion of positive tumor cells was scored as follows: 0 (no positive tumor cells), 1 (<10% positive tumor cells), 2 (10–50% positive tumor cells), 3 (>50% positive tumor cells), and 4 (75–100% positive tumor cells). The staining intensity was graded according to the following criteria: 0 (no staining); 1 (weak staining = light yellow), 2 (moderate staining = yellow brown), and 3 (strong staining = brown). The staining index (SI) was calculated by the product of staining intensity score and proportion of positive tumor cells. Using this method of assessment, the expression of VCAM1 was scored as 0, 1, 2, 3, 4, 6 and 9. An optimal cutoff value was identified: the score of >3 was used to define tumors as high VCAM1 expression, and ≤3 as low expression of VCAM1.

**Wound healing assay, migration assay, invasion assay and cell 3D culture**

For the wound healing assay, cells were seed (2×10^5^ cells/well) in a 6-well plate and incubated for 24 h. A 10 μL sterile pipette tip was used to create a wound in a straight line. Wound healing was imaged daily.

For the migration assay and invasion assay, cells were serum-starved for 24 h and 1×10^5^ cells were plated into the upper chamber of a polycarbonate transwell filter chamber coated without or with Matrigel. After incubation for 24 h, cells inside the chamber were removed using cotton swabs. The migrated cells on the lower membrane surface were fixed in 1% paraformaldehyde and stained with hematoxylin. Migrated cells were counted under a microscope (10 random 100 X fields per well).

For the three-dimensional culture assay, 24-well culture plates were coated with Matrigel. Cells (1×10^5^ cells per well) were seeded incubated for 10 days. A total of five randomly selected fields were chosen to observe the number of invading cells under a microscope.

**Immunofluorescence Assays**

Cells were plated on confocal dish and fixed with paraformaldehyde (4% w:v). After blocking with 0.1% Triton X-100 containing 1% bovine serum albumin in PBS for 1 h, cells were incubated with a primary antibody. The antibodies listed below: cortactin 1:200 (3503, CST, USA) and Alexa 488 goat anti-rabbit 1:200 (ZF-0511, ZSGB-BIO, Beijing). F-actin were stained with Rhodamine Phalloidin 1:1800 (PHDR1, Cytoskeleton, USA). Confocal dishes were counterstained with DAPI (C0060, Solarbio, Beijing) and imaged using a confocal laser-scanning microscope (Olympus FV1000). Data were processed using Adobe Photoshop 7.0 software.

# Supplementary Figures


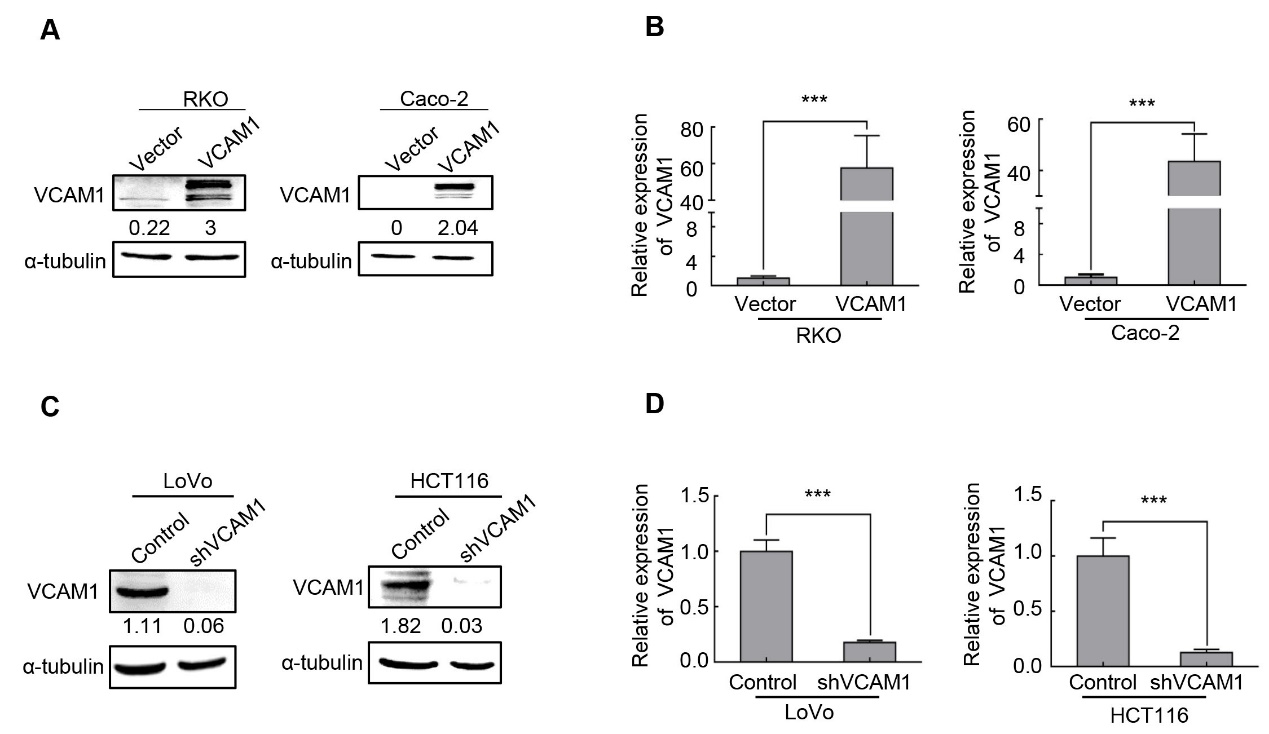


**Supplementary Figure 1.** (A) and (B), Western blotting and Real-time PCR analysis of VCAM1 expression in VCAM1 up-regulated RKO and Caco-2 cells. (C) and (D) Western blotting and Real-time PCR analysis of VCAM1 expression in VCAM1 depleting LoVo and HCT116 cells. Error bars represent mean ± SD calculated from three parallel experiments. ***p* < 0.01, ****p* < 0.001.


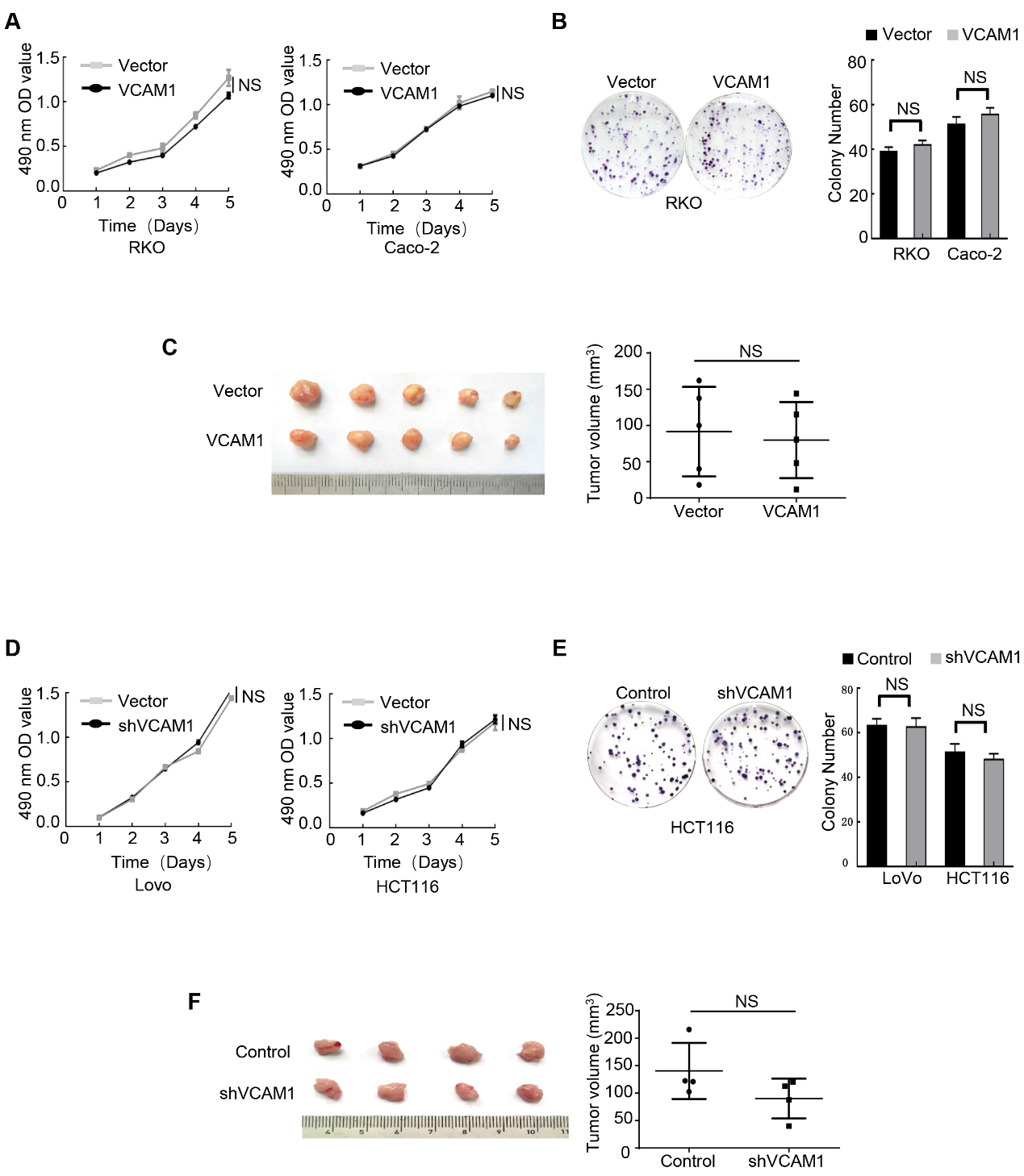


**Supplementary Figure 2.** (A) and (B) Detection of cell proliferation using MTT (factorial analysis) and colony formation assays in the indicated cells with the up-regulated expression of VCAM1. (C) Nude mice Xenograft models by subcutaneously injecting RKO cells with overexpression of VCAM1 were established (n =5). (D) and (E) Effect of cell proliferation in the CRC cells with silencing of VCAM1 via MTT (factorial analysis) and colony formation assays. (F) Subcutaneous xenotransplanted tumor model is operated by injecting HCT116 cells with depletion of VCAM1 (n =4). Error bars represent mean ± SD from three independent experiments. Error bars represent mean ± SD from three independent experiments. NS: *p* > 0.05.


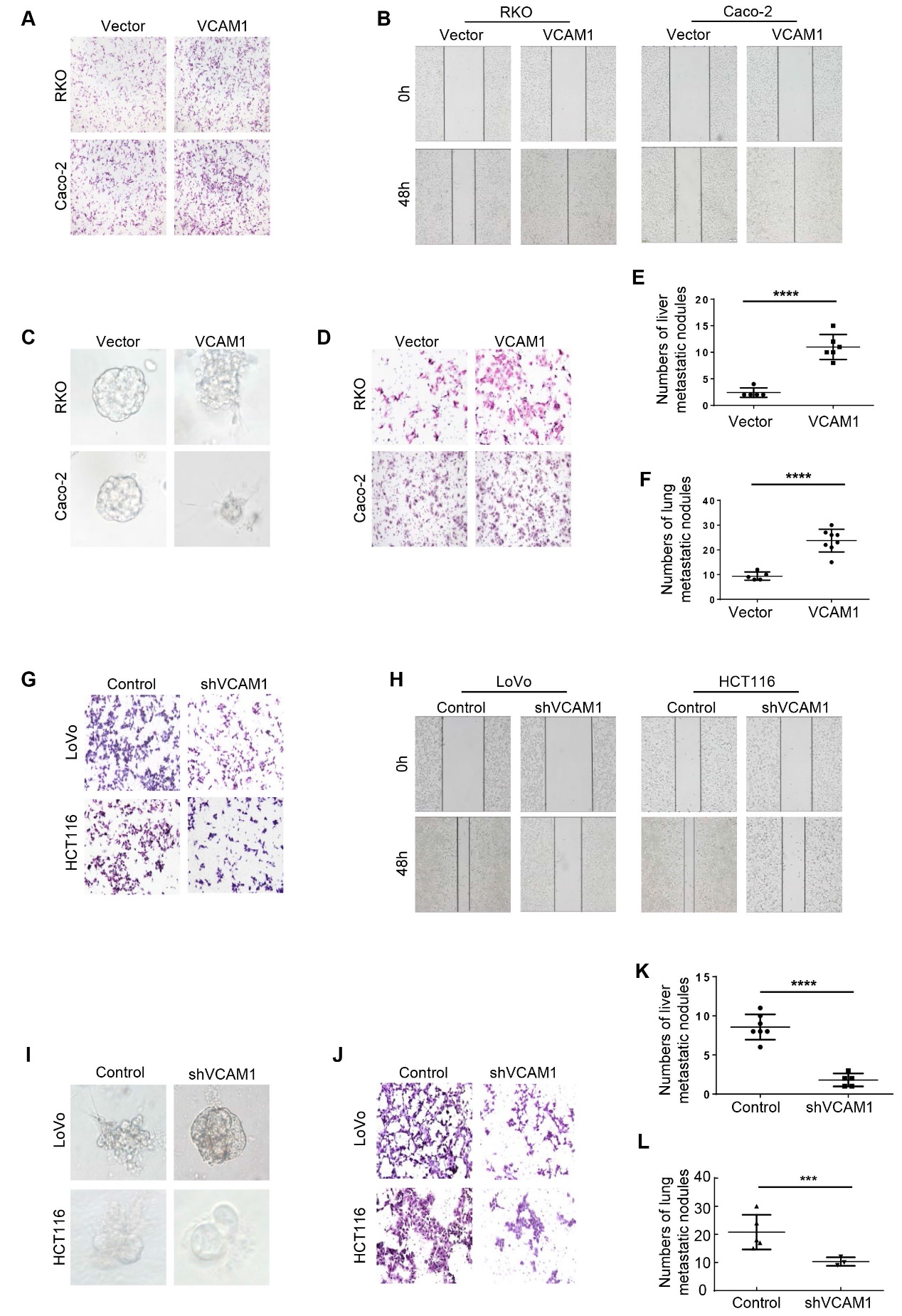


**Supplementary Figure 3.** (A) and (B) Cell migration was determined using Transwell assays and wound-healing assays in the indicated cells. (C) and (D) Cell invasion was detected using 3D cell culture assays and Matrigel-coated Transwell assays (statistic data) in the indicated cells. (E) and (F) Histograms demonstrate the statistics about the numbers of liver metastases and lung metastases nudules in the nude mice (n=5) injected RKO-vector and RKO-VCAM1 cells. (G) and (H) Silencing VCAM1 affected cell migration using transwell assays and wound-healing assays. (I) and (J) Silencing VCAM1 influenced cell invasion using 3D cell culture assays and Matrigel-coated Transwell assays (statistic data). (K) and (J)Histograms demonstrate the statistics about the numbers of liver metastases and lung metastases nodules in the nude mice (n=5) injected with HCT116-vector and HCT116-shVCAM1 cells. Error bars represent mean ± SD from three independent experiments. ****p* < 0.001.


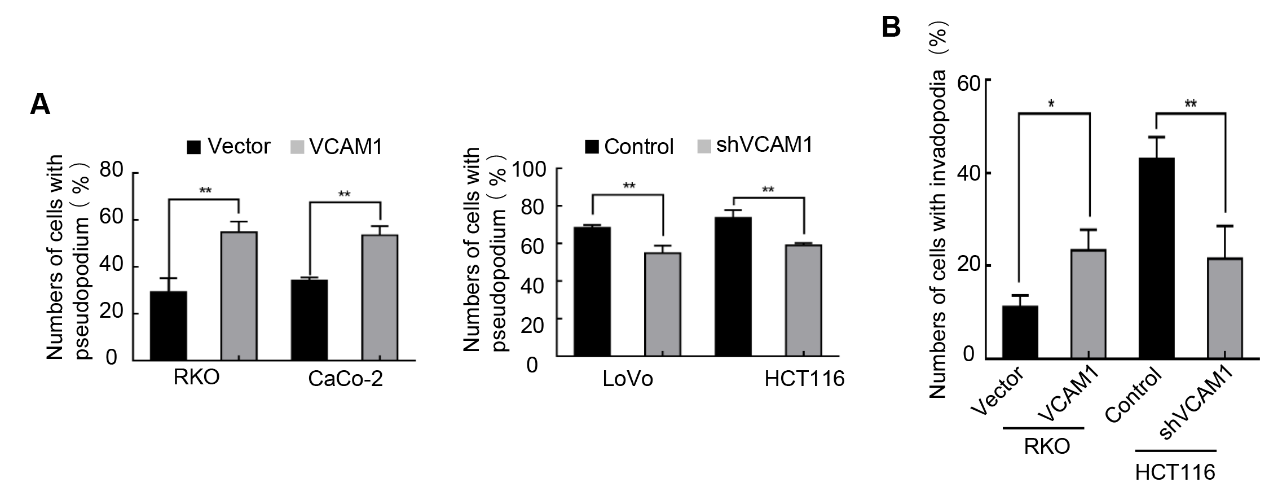


**Supplementary Figure 4.** (A) Quantification of cells with pseudopodia. N= 150 cells/sample. (B) Histogram analysis of numbers of cells with invadopodia. N= 150 cells/sample. **p* < 0.05, ***p* < 0.01.

# 3 Supplementary Tables

**Supplementary Table S1.** Univariate and Multivariate analyses of various prognosis parameters in 123 CRC patients using Cox Regression model

|  | | **Univariate analysis** | | | **Multivariate analysis** | | |
| --- | --- | --- | --- | --- | --- | --- | --- |
| variable | Category | No.  Patients | *P* | S.E | *P* | Relative  Risk | 95%CI |
| VCAM1 | Low | 41 | 0.000 | 0.158 | 0.38 | 1.148 | 0.842-1.566 |
|  | High | 82 |  |  |  |  |  |
| T classification | 0 | 4 | 0.000 | 0.259 | 0.002 | 2.233 | 1.343-3.713 |
|  | 1-2 | 28 |  |  |  |  |  |
|  | 3-4 | 91 |  |  |  |  |  |
| N classification | 0 | 83 | 0.000 | 0.224 | 0.001 | 2.054 | 1.325-3.183 |
|  | 1-2 | 40 |  |  |  |  |  |
| M classification | 0 | 102 | 0.000 | 0.347 | 0.002 | 2.869 | 1.453-5.667 |
|  | 1 | 21 |  |  |  |  |  |

SE: standard error; RR: relative risk; CI: confidence interval
